# Choline PET/CT in the PSMA Era: Clinical Repositioning, Biological Perspectives, and Emerging Applications

**DOI:** 10.3390/diagnostics16132108

**Published:** 2026-07-06

**Authors:** Virginia Rossetti, Lorenzo Fantini, Irene Marini, Monica Celli, Ilaria Grassi, Maddalena Sansovini, Silvia Nicolini, Federica Matteucci, Paola Caroli

**Affiliations:** Nuclear Medicine, IRCCS Istituto Romagnolo per lo Studio dei Tumori “Dino Amadori”, 47014 Meldola, Italy; virginia.rossetti@irst.emr.it (V.R.); lorenzo.fantini@irst.emr.it (L.F.); irene.marini@irst.emr.it (I.M.); monica.celli@irst.emr.it (M.C.); ilaria.grassi@irst.emr.it (I.G.); maddalena.sansovini@irst.emr.it (M.S.); silvia.nicolini@irst.emr.it (S.N.); paola.caroli@irst.emr.it (P.C.)

**Keywords:** choline PET/CT, PSMA, prostate cancer, ^18^F-fluorocholine, primary hyperparathyroidism, molecular imaging

## Abstract

The widespread adoption of prostate-specific membrane antigen (PSMA)-targeted PET/CT has profoundly reshaped molecular imaging in prostate cancer and has substantially reduced the routine use of radiolabeled choline tracers. However, the transition from choline to PSMA imaging should not be interpreted simply as the replacement of one radiopharmaceutical by another, but rather as part of a broader evolution from metabolism-based imaging toward receptor-targeted and biology-driven imaging strategies. This narrative review critically reassesses the residual and emerging role of choline PET/CT in the PSMA era, with particular attention to the biological rationale of choline uptake, selected prostate cancer scenarios, and extra-prostatic applications. In prostate cancer, PSMA PET/CT remains the dominant imaging modality because of its superior diagnostic performance, particularly in biochemical recurrence; nevertheless, choline PET/CT may provide complementary metabolic information in highly selected settings, including PSMA-low or heterogeneous disease, aggressive or dedifferentiated variants, neuroendocrine transformation, equivocal PSMA findings, and limited PSMA availability. These prostate cancer applications, however, are supported mainly by biological rationale, indirect evidence, and limited clinical data and should therefore be regarded as exploratory rather than established indications. By contrast, ^18^F-fluorocholine PET/CT has emerged as a clinically established imaging modality in primary hyperparathyroidism, particularly after negative or inconclusive conventional imaging, with prospective studies and meta-analyses demonstrating high detection rates and superior performance compared with conventional scintigraphic techniques. Additional applications in hepatocellular carcinoma and selected neuro-oncologic settings remain exploratory and require further validation. Overall, choline PET/CT should not be considered obsolete in the PSMA era, but selectively repositioned within biology-driven and multiparametric imaging strategies, with its strongest evidence currently supporting primary hyperparathyroidism and its other applications requiring cautious interpretation and further prospective validation.

## 1. Introduction

Over the last decade, molecular imaging in oncology has undergone a profound transformation, particularly in prostate cancer, where the development of prostate-specific membrane antigen (PSMA)-targeted positron emission tomography/computed tomography (PET/CT) has dramatically reshaped diagnostic algorithms and clinical decision-making. Owing to its high tumor-to-background contrast and superior detection rates, especially in biochemical recurrence at low prostate-specific antigen (PSA) levels, PSMA PET/CT has progressively become the reference molecular imaging modality in several clinical settings and has largely replaced choline PET/CT in most routine prostate cancer imaging settings [1,2,3].

Before the widespread adoption of PSMA-targeted imaging, radiolabeled choline tracers represented one of the most extensively used PET radiopharmaceuticals in prostate cancer imaging. By reflecting phospholipid membrane synthesis and cellular proliferation, choline PET/CT provided clinically meaningful information for disease localization, staging, and restaging, particularly in patients with biochemical recurrence [4,5,6].

However, the transition from choline to PSMA imaging should not be interpreted simply as the replacement of a less sensitive tracer by a superior one. Rather, it reflects a broader evolution from metabolism-based imaging toward receptor-targeted and biology-driven imaging strategies [7,8]. Increasing evidence suggests that prostate cancer remains biologically heterogeneous, with variable PSMA expression across different disease states and tumor phenotypes, particularly in advanced, aggressive, or treatment-resistant disease [9,10]. At the same time, the role of choline PET/CT has progressively expanded beyond prostate cancer. Among extra-prostatic applications, ^18^F-fluorocholine PET/CT has emerged as a clinically established imaging modality in primary hyperparathyroidism, particularly after negative or inconclusive conventional imaging [11,12,13].

Therefore, in the PSMA era, choline PET/CT should no longer be viewed as an obsolete imaging modality but rather as a selectively repositioned tracer with residual, complementary, and emerging clinical applications within modern biology-driven imaging pathways.

**Figure 1:**
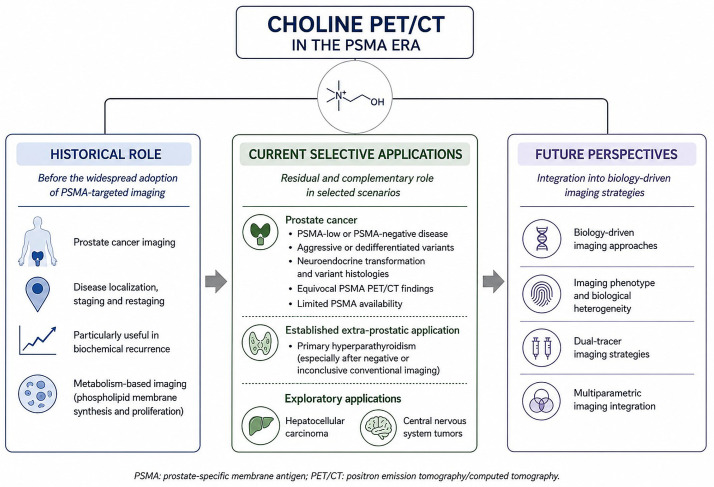
Repositioning of choline PET/CT in the PSMA era. The figure summarizes the evolution of choline PET/CT from its historical role in prostate cancer imaging toward selected residual and emerging applications. In prostate cancer, choline PET/CT may retain a complementary role in selected scenarios characterized by PSMA-low or heterogeneous disease, aggressive or dedifferentiated variants, neuroendocrine transformation, equivocal PSMA findings, or limited PSMA availability. Beyond prostate cancer, primary hyperparathyroidism represents the most established extra-prostatic application, while hepatocellular carcinoma and central nervous system tumors remain exploratory indications. Future perspectives include biology-driven imaging, imaging phenotype assessment, dual-tracer strategies, and multiparametric imaging integration. Figure created with the support of artificial intelligence-assisted image generation and subsequently reviewed, modified, and approved by the authors.

The progressive clinical and biological repositioning of choline PET/CT in the PSMA era is schematically illustrated in Figure 1.

## 2. From Choline to PSMA: A Paradigm Shift

The clinical transition from choline PET/CT to PSMA-targeted imaging represents one of the most significant changes in prostate cancer molecular imaging. While choline PET/CT provided whole-body metabolic information and played an important role in the pre-PSMA era, particularly in biochemical recurrence, its diagnostic limitations became increasingly evident in patients with low PSA values and small-volume disease [4,5,6].

PSMA PET/CT has substantially changed this scenario by providing higher lesion detectability, improved tumor-to-background contrast, and greater sensitivity for nodal and distant metastatic disease [1,2,3]. These advantages explain why PSMA-targeted imaging has rapidly become the preferred molecular imaging approach in most prostate cancer settings [8].

However, this paradigm shift should not be reduced to a simple hierarchy of tracer performance. Choline and PSMA tracers interrogate different biological processes: choline uptake reflects membrane metabolism and cellular proliferation [4,7], whereas PSMA-targeted tracers reflect receptor expression [8]. These biological pathways may not evolve in parallel, particularly in advanced, heterogeneous, or treatment-resistant disease [9,10].

Accordingly, the relevant question in the contemporary PSMA era is no longer whether choline PET/CT can compete with PSMA PET/CT in routine prostate cancer imaging. Rather, it is whether choline PET/CT may still provide complementary metabolic information in selected clinical and biological contexts, especially when receptor-targeted imaging does not fully reflect disease phenotype.

## 3. Residual Role of Choline PET/CT in Prostate Cancer

Although PSMA-targeted PET imaging has become the dominant molecular imaging approach in prostate cancer, receptor-targeted imaging alone may not fully capture the biological complexity of selected advanced or treatment-resistant disease. PSMA PET/CT has demonstrated superior diagnostic performance in most standard clinical scenarios and is now incorporated into international recommendations and standardized reporting systems [1,2,3,8,14,15]. Nevertheless, increasing evidence suggests that PSMA expression may be heterogeneous, particularly in advanced, aggressive, or treatment-resistant prostate cancer [9,10]. In this context, the potential residual role of choline PET/CT should be interpreted as complementary and highly selective, rather than as an alternative to PSMA-targeted imaging in routine clinical practice.

The residual and emerging applications of choline PET/CT discussed in this review, together with their underlying biological rationale and relative strength of evidence, are summarized in Table 1.

Although PSMA PET/CT represents the dominant molecular imaging modality in prostate cancer, it has important limitations that should be acknowledged. PSMA expression may be low, heterogeneous, or absent in selected disease phenotypes, particularly in advanced, dedifferentiated, neuroendocrine, or treatment-resistant prostate cancer. In addition, false-negative findings may occur in small-volume disease below PET spatial resolution, while equivocal uptake related to benign, inflammatory, traumatic, ganglionic, or treatment-related changes may reduce diagnostic confidence. Therefore, PSMA PET/CT should not be considered a universal representation of prostate cancer biology. In these settings, complementary imaging approaches may be useful, but their clinical role depends on the specific biological question: FDG PET/CT is the more established metabolic tracer for aggressive or dedifferentiated disease, whereas choline PET/CT has a more limited and selective role in prostate cancer.

### 3.1. PSMA-Negative or Low-PSMA Disease

Despite the excellent diagnostic performance of PSMA-targeted imaging, a subset of prostate cancers may demonstrate low, heterogeneous, or absent PSMA expression, particularly in advanced, aggressive, or treatment-resistant disease [9,10]. However, PSMA-negative or non-diagnostic findings should not be interpreted as a single biological entity. They may reflect true low or absent PSMA expression, tumor dedifferentiation, neuroendocrine transformation, inter- and intra-lesion heterogeneity, small-volume disease below the spatial resolution of PET imaging, or technical and interpretative factors.

The prevalence of clinically relevant PSMA-negative disease varies according to disease stage, patient selection, imaging criteria, and the tracer used. In advanced metastatic castration-resistant prostate cancer, discordant imaging phenotypes, particularly FDG-positive/PSMA-negative lesions, have been associated with aggressive biology and poorer outcomes. However, most of the available evidence concerns FDG/PSMA discordance rather than choline/PSMA discordance and cannot be directly extrapolated to support a routine role for choline PET/CT.

Unlike PSMA-targeted tracers, choline uptake reflects phospholipid membrane synthesis and cellular proliferation rather than receptor expression [4,7]. On this basis, choline PET/CT may theoretically provide complementary metabolic information in selected patients with persistent clinical suspicion and negative or non-diagnostic PSMA PET/CT findings. Nevertheless, this potential role is currently supported mainly by biological rationale and limited indirect evidence. Robust prospective studies specifically evaluating choline PET/CT in PSMA-negative or PSMA-low disease are lacking.

Therefore, choline PET/CT should not be proposed as a routine diagnostic step after negative PSMA PET/CT. Its use in this setting should be considered exploratory and limited to highly selected cases, ideally within multidisciplinary discussion or research-oriented imaging strategies.

### 3.2. Aggressive and Dedifferentiated Variants

Prostate cancer encompasses a broad biological spectrum, ranging from indolent androgen-sensitive disease to highly aggressive and poorly differentiated phenotypes. As tumor biology evolves, imaging characteristics may also change substantially.

Aggressive or dedifferentiated variants may exhibit heterogeneous or reduced PSMA uptake despite clinically significant disease burden [9,10]. In this setting, metabolism-based imaging may provide information that is not entirely captured by receptor-targeted tracers. Although most available evidence concerns FDG imaging rather than choline PET/CT, the same conceptual framework supports the potential value of complementary metabolic imaging in biologically aggressive disease [9].

Accordingly, choline PET/CT may be considered a potential adjunctive imaging tool in selected cases characterized by discordance between clinical aggressiveness and PSMA-targeted imaging findings. Nevertheless, available evidence remains limited, and this role should currently be regarded as exploratory.

### 3.3. Neuroendocrine Transformation and Androgen-Independent Disease

Neuroendocrine transformation represents one of the most aggressive forms of prostate cancer progression and is frequently associated with reduced androgen receptor signaling and heterogeneous or low PSMA expression [9,10]. From an imaging perspective, this phenotype may result in reduced sensitivity of PSMA-targeted imaging and greater reliance on phenotype-oriented or metabolism-based imaging approaches.

Because choline uptake reflects membrane metabolism rather than receptor expression, choline PET/CT may theoretically provide complementary information in selected PSMA-low or PSMA-negative lesions [4,7]. However, evidence specifically supporting choline PET/CT in neuroendocrine prostate cancer remains scarce. Therefore, this potential application should currently be interpreted primarily as a biological rationale rather than as a validated clinical indication.

### 3.4. Equivocal PSMA Findings and Problem-Solving Scenarios

Equivocal PSMA findings remain a relevant issue in clinical practice. Mild or nonspecific uptake, inflammatory conditions, benign lesions, treatment-related changes, ganglia, fractures, and atypical lesion distribution may occasionally complicate image interpretation and reduce diagnostic confidence [8,14,15,16,17]. Standardized reporting systems, such as E-PSMA and PSMA-RADS, have been developed to improve interpretation and communication of uncertain findings [14,15].

In highly selected cases, choline PET/CT may provide complementary metabolic information as a problem-solving tool. Discordant findings between PSMA-targeted and metabolism-based imaging may help refine interpretation, particularly in patients with biochemical progression, aggressive clinical features, or persistent clinical suspicion despite non-definitive PSMA PET/CT findings. However, the use of choline PET/CT in this setting should be individualized and cannot currently be considered a routine diagnostic strategy.

### 3.5. Limited PSMA Availability and Regulatory Issues

Despite the rapid global expansion of PSMA-targeted imaging and its incorporation into contemporary guidelines and procedure standards, access to PSMA PET/CT may remain heterogeneous across countries and healthcare systems [3,8]. Regulatory approval, reimbursement policies, tracer production, local infrastructure, and economic constraints may influence its implementation in routine clinical practice.

In several institutions, choline PET/CT may still represent a pragmatic imaging option, particularly in healthcare systems where access to PSMA-targeted imaging is limited or not consistently available. This residual role should not be interpreted as evidence of biological superiority, but rather as a real-world indication driven by logistical, regulatory, and economic factors.

### 3.6. FDG Versus Choline PET/CT as Complementary Metabolic Imaging

When discussing complementary metabolic imaging in the PSMA era, FDG PET/CT represents the most established comparator, particularly in advanced, aggressive, dedifferentiated, or neuroendocrine prostate cancer [9,18,19]. FDG uptake reflects glucose metabolism and is more frequently associated with aggressive tumor biology, treatment resistance, and poorer prognosis. In metastatic castration-resistant prostate cancer, FDG-positive/PSMA-negative lesions have been increasingly recognized as a clinically relevant discordant phenotype and may identify patients with biologically aggressive disease [9,10]. Consistently, the TheraP trial required both PSMA PET/CT and FDG PET/CT for eligibility assessment and excluded patients with discordant FDG-positive/PSMA-negative lesions, supporting the clinical relevance of FDG as a complementary metabolic tracer in advanced prostate cancer [18].

By contrast, choline PET/CT reflects phospholipid membrane synthesis and cellular proliferation rather than glycolytic metabolism [4,7]. Historically, this mechanism supported the use of choline PET/CT in biochemical recurrence and restaging of prostate cancer. However, in the contemporary PSMA era, choline PET/CT has substantially less evidence than FDG PET/CT for the characterization of aggressive PSMA-negative or dedifferentiated disease. Therefore, choline PET/CT should not be considered interchangeable with FDG PET/CT in this setting.

In practical terms, FDG PET/CT may be more appropriate when the clinical question concerns aggressive biology, suspected dedifferentiation, neuroendocrine transformation, or discordant progression despite low or heterogeneous PSMA expression [9,10]. Choline PET/CT may provide complementary information only in selected circumstances, particularly where prior choline-based imaging is available, where local tracer availability influences imaging pathways, or when membrane-metabolism imaging is considered relevant within a multidisciplinary assessment. Nevertheless, this remains a selective and insufficiently validated application in prostate cancer.

The distinction is different outside prostate cancer. In primary hyperparathyroidism, ^18^F-fluorocholine PET/CT has a specific and clinically established role for the localization of hyperfunctioning parathyroid tissue, whereas FDG PET/CT is not considered a relevant competing tracer for this indication [11,12,13].

## 4. Beyond Prostate Cancer

Although prostate cancer historically represented the principal field of application for choline PET/CT, the inclusion of extra-prostatic applications in this review is intended to clarify how choline PET/CT has been clinically repositioned beyond its original onco>logic use. These indications are not equivalent in terms of evidence or clinical maturity. Primary hyperparathyroidism is discussed because it currently represents the most established and evidence-supported extra-prostatic application of ^18^F-fluorocholine PET/CT [11,12,13,20,21,22,23,24,25,26]. Hepatocellular carcinoma, by contrast, is included as an exploratory oncologic model in which FDG and choline uptake may reflect different aspects of tumor differentiation and metabolic heterogeneity [27,28,29,30,31,32,33].

### 4.1. Hyperparathyroidism

Primary hyperparathyroidism has become one of the most relevant non-oncologic applications of choline PET/CT. Accurate preoperative localization of hyperfunctioning parathyroid tissue is essential to guide focused or minimally invasive parathyroidectomy, reduce operative time, and increase the likelihood of successful surgical treatment. Conventional imaging usually relies on neck ultrasonography and ^99m^Tc-sestamibi scintigraphy, often combined with SPECT/CT; however, these techniques may show limited performance in patients with small adenomas, ectopic glands, multiglandular disease, coexisting thyroid abnormalities, previous neck surgery, or discordant imaging findings [18,21,23].

In this context, ^18^F-fluorocholine PET/CT has demonstrated excellent diagnostic performance for the localization of hyperfunctioning parathyroid tissue. In a prospective study including 103 patients with primary hyperparathyroidism, Cuderman et al. reported a sensitivity of 92% for ^18^F-fluorocholine PET/CT, compared with 39–56% for individual conventional scintigraphic methods and 65% for combined conventional imaging. In patients with multiple hyperfunctioning glands, ^18^F-fluorocholine PET/CT showed a sensitivity of 88%, whereas conventional imaging showed substantially lower sensitivity, ranging from 22% to 34% for individual techniques and 44% when combined [11].

Similarly, in a prospective dual-center study of 100 patients, Beheshti et al. reported a patient-based detection rate of 93% for ^18^F-fluorocholine PET/CT compared with 61% for ^99^mTc-MIBI/tetrofosmin SPECT/CT. In lesion-based analysis, ^18^F-fluorocholine PET/CT showed sensitivity, specificity, positive predictive value, negative predictive value, and accuracy of 93.7%, 96.0%, 90.2%, 97.4%, and 95.3%, respectively, compared with 60.8%, 98.5%, 94.1%, 86.3%, and 87.7% for conventional SPECT/CT imaging [19].

The added value of ^18^F-fluorocholine PET/CT is particularly evident in patients with negative or inconclusive conventional imaging and in challenging pre-surgical localization scenarios. Grimaldi et al. specifically highlighted the clinical utility of ^18^F-fluorocholine PET/CT in difficult cases of primary hyperparathyroidism, supporting its role as a problem-solving tool when conventional imaging is insufficient [20]. In the prospective APACH1 study, which enrolled patients with primary hyperparathyroidism and negative or inconclusive cervical ultrasound and ^99^mTc-sestamibi SPECT/CT, ^18^F-fluorocholine PET/CT achieved per-lesion and per-patient sensitivities of 91.3% and 90.5%, respectively, with corresponding positive predictive values of 87.5% and 86.4%. Moreover, ^18^F-fluorocholine PET/CT guided surgery in 88% of patients and allowed bilateral cervical exploration to be avoided in the majority of cases [22].

These findings are supported by systematic reviews and meta-analyses reporting consistently high diagnostic performance of choline PET imaging for the detection of hyperfunctioning parathyroid glands [12,13]. The high spatial resolution of PET imaging, favorable lesion-to-background contrast, and relatively short acquisition protocols represent important technical and practical advantages of ^18^F-fluorocholine PET/CT [11,13,20,21,23].

Current international recommendations generally position ^18^F-fluorocholine PET/CT as a second-line or problem-solving modality after negative or inconclusive conventional imaging [21,23]. However, increasing evidence and expert opinion support its potential use as a first-line imaging modality in selected centers with appropriate expertise, particularly when minimally invasive surgery is planned [12,13,23]. Therefore, primary hyperparathyroidism represents the most clinically established and evidence-supported extra-prostatic application of ^18^F-fluorocholine PET/CT and provides a paradigmatic example of how choline-based imaging has evolved from a predominantly oncologic imaging modality into a multidisciplinary molecular imaging tool with established clinical utility beyond prostate cancer [12,21,23].

### 4.2. Hepatocellular Carcinoma

Hepatocellular carcinoma (HCC) is characterized by marked biological and metabolic heterogeneity, which may partly explain the variable performance of different PET tracers. FDG PET/CT generally shows higher uptake in poorly differentiated and biologically aggressive tumors, whereas choline PET/CT tends to show higher uptake in well- and moderately differentiated HCC, reflecting differences in glucose metabolism and membrane lipid synthesis [27,28,29,30].

Early comparative studies supported the complementary value of these tracers. Yamamoto et al. reported that ^11^C-choline PET detected HCC lesions with higher sensitivity than FDG PET, particularly in well-differentiated tumors [27]. In another prospective study including patients with chronic liver disease and suspected HCC, the sensitivity for HCC detection was 75% for ^18^F-fluorocholine PET/CT, 36% for FDG PET/CT, and 93% when both tracers were combined, suggesting that dual-tracer imaging may improve lesion detection compared with either tracer alone [29].

However, the relationship between tracer uptake and tumor differentiation is not absolute. In a systematic review of dual ^18^F-FDG/^18^F-choline PET behavior in histology-proven HCC, Ghidaglia et al. included 6 studies comprising 99 HCC lesions. Among well-differentiated HCCs, 51% were positive only for ^18^F-choline and 39% were positive for both ^18^F-FDG and ^18^F-choline. Among less-differentiated tumors, 37% were positive only for FDG, 36% were positive for both tracers, and 25% were positive only for ^18^F-choline. These data indicate substantial overlap between uptake patterns and histological differentiation, limiting the use of dual-tracer PET as a stand-alone surrogate marker of tumor differentiation [28].

More recently, Sönmez et al. evaluated the clinical utility of dual ^18^F-FDG/^18^F-choline PET/CT in a tertiary-center cohort of 168 HCC patients. Dual PET/CT findings increased the BCLC stage in 21 patients (12.5%) and modified the treatment strategy in 41 patients (24.4%). In addition, combined PET parameters and alpha-fetoprotein levels were associated with disease-free survival, suggesting a potential role for dual-tracer PET/CT in refining risk stratification and treatment planning in selected patients [30].

Overall, these findings support the concept that FDG and choline PET/CT may provide complementary biological information in HCC. Nevertheless, the available evidence remains limited, heterogeneous, and insufficient to support routine clinical use. Therefore, choline PET/CT in HCC should be regarded as an exploratory and selective tool for biological characterization and multiparametric assessment rather than as a standard imaging modality.

### 4.3. Central Nervous System Tumors

In neuro-oncology, choline PET/CT has mainly been investigated in small and selected studies as a complementary imaging modality integrated with MRI, rather than as a standard imaging approach. Because choline uptake reflects membrane turnover and cellular proliferation, it may provide additional metabolic information in selected brain tumors and in challenging post-treatment settings [7,31,32,33].

One of the most relevant potential applications is the differentiation between tumor recurrence and radiation necrosis in previously treated gliomas or brain tumors, where conventional MRI may be inconclusive. Gao et al. reported the diagnostic accuracy of ^11^C-choline PET in differentiating glioma recurrence from radiation necrosis in a systematic review and meta-analysis [32]. Data from Takenaka et al. also supported the potential utility of ^11^C-choline PET for differentiating recurrent glioma from radiation necrosis after radiotherapy, although ^11^C-methionine PET showed superior diagnostic performance [33].

Choline PET/CT has also been explored for the characterization of selected intracranial lesions, including MRI-suspected low-grade gliomas, although available evidence remains limited and is generally based on small studies or heterogeneous patient populations [31,32,33].

Nevertheless, amino acid PET tracers remain the preferred molecular imaging agents in several neuro-oncologic settings because of their more favorable physiological brain background and stronger evidence base, as reflected by joint EANM/EANO/RANO/SNMMI practice guidelines for PET imaging of gliomas [34]. Consequently, choline PET/CT should not be regarded as a primary neuro-oncologic imaging modality, but rather as an adjunctive and highly selective tool when alternative tracers are unavailable or when additional metabolic information may be clinically useful.

### 4.4. Other Emerging Indications

Additional exploratory applications of choline PET/CT have been described in selected malignancies and incidental findings, further supporting the concept of choline imaging as a flexible metabolism-based imaging modality. However, most of these indications remain supported only by limited evidence, preliminary experiences, or isolated clinical reports.

Overall, the diversification of molecular imaging strategies suggests that future imaging pathways may progressively evolve toward multiparametric and biology-oriented approaches. Within this framework, choline PET/CT may retain value not as a universal tracer, but as a selective modality capable of providing complementary metabolic information in specific clinical and biological contexts.

## 5. Biological and Metabolic Perspectives

The transition from choline PET/CT to PSMA-targeted imaging reflects a broader evolution in oncologic molecular imaging, moving from metabolism-based approaches toward receptor-targeted and phenotype-oriented imaging strategies [1,7,8,35,36,37,38,39].

Radiolabeled choline tracers primarily reflect phospholipid membrane synthesis and cellular proliferation [4,7], whereas PSMA-targeted tracers evaluate receptor expression [8]. Consequently, metabolism-based imaging and receptor-targeted imaging may provide partially different information regarding tumor phenotype and disease biology.

This concept is particularly relevant in advanced and biologically heterogeneous prostate cancer, where receptor expression, metabolic activity, dedifferentiation, and treatment-related changes may not evolve in parallel [9,10,35,36]. In this setting, discordant uptake patterns across different tracers may reflect underlying biological heterogeneity rather than simple technical differences.

The growing recognition of imaging heterogeneity has contributed to the concept of an “imaging phenotype”, whereby different radiopharmaceuticals may capture distinct molecular, metabolic, or functional characteristics of the same disease. Although this approach remains under investigation, it is increasingly relevant within precision oncology, where imaging is expected not only to localize disease but also to contribute to biological characterization and treatment selection [35,36,37,38,39].

Within this evolving framework, choline PET/CT should not be interpreted as competing with PSMA PET/CT, but rather as a potential source of complementary metabolic information in selected clinical scenarios. This concept is consistent with other oncologic models in which multiple tracers are used to characterize different aspects of tumor biology, such as the complementary role of FDG and choline imaging in hepatocellular carcinoma [27,28,29,30].

Overall, the PSMA era has reinforced the importance of biology-driven imaging rather than eliminating the value of metabolism-based tracers. The future role of choline PET/CT will likely depend on its ability to contribute selectively to multiparametric imaging strategies in patients whose disease biology is not fully represented by receptor-targeted imaging alone.

## 6. Future Perspectives

Dual-tracer approaches combining receptor-targeted and metabolism-based radiopharmaceuticals may help characterize tumor heterogeneity and identify aggressive or biologically discordant disease phenotypes [9,10,35,36]. This concept is also supported by other oncologic models, such as hepatocellular carcinoma, in which FDG and choline PET/CT may provide complementary information on tumor biology [27,28,29,30]. In prostate cancer, this approach may be particularly relevant in advanced or treatment-resistant disease, where receptor expression and metabolic activity may not evolve in parallel [9,10,35,36].

Beyond visual interpretation, quantitative imaging approaches may further refine the clinical value of PET/CT. Radiomics and advanced computational tools may support the extraction of imaging features related to tumor heterogeneity, tracer distribution, and treatment response, potentially contributing to more personalized diagnostic and therapeutic pathways [37,38,39].

However, these approaches remain investigational and require prospective validation, methodological standardization, and harmonization of imaging protocols before routine clinical implementation.

Future molecular imaging strategies will likely become increasingly phenotype-oriented and multiparametric, integrating metabolic, receptor-targeted, and functional information according to the specific clinical question, disease stage, and therapeutic context.

## 7. Conclusions

The widespread adoption of PSMA-targeted imaging has profoundly reshaped the role of choline PET/CT in contemporary molecular imaging. Although PSMA PET/CT has become the dominant imaging modality in prostate cancer because of its superior diagnostic performance, the transition from choline to PSMA imaging should not be interpreted simply as the replacement of one tracer by another.

Increasing evidence highlights the biological heterogeneity of advanced prostate cancer and the potential limitations of relying exclusively on receptor-targeted imaging. In selected scenarios, including PSMA-low or heterogeneous disease, aggressive variants, neuroendocrine transformation, equivocal PSMA findings, and limited tracer availability, choline PET/CT may still provide complementary metabolic information. However, these applications remain selective and are currently supported mainly by biological rationale, indirect evidence, and limited clinical data.

At the same time, ^18^F-fluorocholine PET/CT has emerged as a clinically relevant imaging modality in primary hyperparathyroidism, where evidence supporting its diagnostic performance is substantially stronger and increasingly consolidated.

Overall, the PSMA era has not eliminated the role of choline PET/CT, but rather contributed to its clinical and biological repositioning. Future molecular imaging strategies will likely become increasingly biology-driven and multiparametric, integrating receptor-targeted, metabolic, and functional information to better reflect disease complexity and heterogeneity.

## Figures and Tables

**Table 1:** Residual and emerging applications of choline PET/CT in the PSMA era, including their rationale, potential clinical role, and current evidence level/clinical maturity.

Clinical Setting	Rationale	Potential Clinical Role	Current Evidence/Clinicalmaturity
**PSMA-low/heterogeneous prostate cancer**	Variable or reduced PSMA expression; possible biological, technical, or lesion-size-related causes of PSMA negativity	Exploratory complementary metabolic imaging in highly selectedcases; not a routine diagnostic step after negative PSMAPET/CT	Low/hypothesis-generating
**Aggressive/dedifferentiatedprostate cancer variants**	Tumor heterogeneity, dedifferentiation, and increasedmetabolic activity; evidence mainly extrapolated fromFDG/PSMA discordance	Exploratory adjunctive characterization of aggressive disease;FDG PET/CT remains the more established metaboliccomparator	Low/indirect evidence
**Neuroendocrine transformation**	Reduced or heterogeneous PSMA expression with possiblepreserved metabolic activity	Potential complementary imaging approach in selected cases;not validated as a routine clinical indication	Low/exploratory
**Equivocal PSMA findings**	Discordant or uncertain receptor-targeted imaging findings	Problem-solving adjunctive imaging in highly selected cases, ideally within multidisciplinary assessment	Low
**Neuroendocrine transformation**	Context-dependent rationale: regulatory, logistical, economic, or tracer-availability constraints	Pragmatic alternative imaging option when PSMA PET/CT is notavailable or not consistently accessible	Context-dependent
**Limited PSMA availability**	High ^18^F-fluorocholine uptake in hyperfunctioningparathyroid glands Metabolic heterogeneity and complementary uptake patternswith FDG	Established clinical application, particularly after negative orinconclusive conventional imaging	High
**Primary hyperparathyroidism**	Metabolic heterogeneity and complementary uptake patternswith FDG	Exploratory dual-tracer characterization of tumor phenotype andbiological aggressiveness	Low-Moderate/exploratory
**Central nervous system tumors**	Metabolic characterization of selected lesions and post-treatment changes	Adjunctive imaging modality in selected cases when standardimaging is inconclusive or alternative tracers are unavailable	Low/exploratory

Note: Evidence level and clinical maturity were assigned as a narrative appraisal rather than as a formal GRADE assessment. High indicates applications supported by prospective studies, systematic reviews, and/or meta-analyses, consistent diagnostic performance, and partial or established integration into clinical recommendations. Low–Moderate indicates applications supported by limited prospective or retrospective studies and biologically plausible evidence, but with heterogeneous data and no routine clinical implementation. Low indicates applications mainly supported by biological rationale, small observational studies, indirect evidence, or hypothesis-generating data. Context-dependent indicates settings in which the role of choline PET/CT is driven primarily by regulatory, logistical, economic, or tracer-availability factors rather than by evidence of biological or diagnostic superiority.

## Data Availability

No new data were created or analyzed in this study. Data sharing is not applicable to this article.
